# Experimental Aspects Suggesting a “Fluxus” of Information in the Virions of Herpes Simplex Virus Populations

**DOI:** 10.3389/fmicb.2017.02625

**Published:** 2017-12-22

**Authors:** Luis A. Scolaro, Julieta S. Roldan, Clara Theaux, Elsa B. Damonte, Maria J. Carlucci

**Affiliations:** ^1^Laboratorio de Virología, Departamento de Química Biológica, Facultad de Ciencias Exactas y Naturales, Universidad de Buenos Aires, Buenos Aires, Argentina; ^2^Instituto de Química Biológica de la Facultad de Ciencias Exactas y Naturales, Consejo Nacional de Investigaciones Científicas y Técnicas, Universidad de Buenos Aires, Buenos Aires, Argentina

**Keywords:** herpes simplex virus, virus–host interactions, microRNAs, non-coding RNAs, regulatory networks, epigenetic, viral population, carrageenans

## Abstract

Our perspective on nature has changed throughout history and at the same time has affected directly or indirectly our perception of biological processes. In that sense, the “fluxus” of information in a viral population arises a result of a much more complex process than the encoding of a protein by a gene, but as the consequence of the interaction between all the components of the genome and its products: DNA, RNA, and proteins and its modulation by the environment. Even modest “agents of life” like viruses display an intricate way to express their information. This conclusion can be withdrawn from the huge quantity of data furnished by new and potent technologies available now to analyze viral populations. Based on this premise, evolutive processes for viruses are now interpreted as a simultaneous and coordinated phenomenon that leads to global (i.e., not gradual or ‘random’) remodeling of the population. Our system of study involves the modulation of herpes simplex virus populations through the selective pressure exerted by carrageenans, natural compounds that interfere with virion attachment to cells. On this line, we demonstrated that the passaging of virus in the presence of carrageenans leads to the appearance of progeny virus phenotipically different from the parental seed, particularly, the emergence of syncytial (syn) variants. This event precedes the emergence of mutations in the population which can be readily detected five passages after from the moment of the appearance of syn virus. This observation can be explained taking into consideration that the onset of phenotypic changes may be triggered by “environmental-sensitive” glycoproteins. These “environmental-sensitive” glycoproteins may act by themselves or may transmit the stimulus to “adapter” proteins, particularly, proteins of the tegument, which eventually modulate the expression of genomic products in the “virocell.” The modulation of the RNA network is a common strategy of the virocell to respond to environmental changes. This “fast” adaptive mechanism is followed eventually by the appearance of mutations in the viral genome. In this paper, we interpret these findings from a philosophical and scientific point of view interconnecting epigenetic action, exerted by carragenans from early RNA network–DNA interaction to late DNA mutation. The complexity of HSV virion structure is an adequate platform to envision new studies on this topic that may be complemented in a near future through the analysis of the genetic dynamics of HSV populations.

## Background

Currently, the study of biological processes, not in the understanding of the process as a whole but in the fragmented analysis increasingly smaller and dazzled by the new technologies, allow us to have a large amount of data that has generated a crisis due to excessive information. Metagenomics studies are a good example of this fact. Such information is lacking in organization and meaningful understanding within the conceptual paradigms of biological phenomena and, therefore, in the interpretation of the data available to us within a general context (Sandín, 2004). A problem that has its origin in the lack of consistency of the theoretical base of biology, this means, in the explanation of the phenomena of life. As explain Oltvai and Barabási (2002) at present, it is widely accepted that DNA is not the only container of biological complexity. The genome, transcriptome, proteome, and metabolome represent distinct levels of organization at which information can be stored and processed. Also, various cellular programs reside at these levels. Thus, although the genome almost exclusively stores long-term information, the proteome is essential for storing information in the short term and the recovery of this information is controlled by transcription factors strongly influenced by the metabolome (Bray, 1995). These different levels of organization and cellular functionality constitute groups of heterogeneous components that would act all interconnected in large networks (Oltvai and Barabási, 2002). Thus, the integration of complex systems would imply that the complexity of life phenomena derives from a great initial complexity of their constituent units (i.e., not only key agents of DNA replication, etc.) and that the properties of the systems that make up life (cells, organs, organisms, ecosystems) are a consequence of the properties of its components (on the other hand, with extremely conserved processes). Populations of viruses are also modeled in this way by processes that take place in the “virocell,” an infected cell whose aim is to produce virions. In this line, viruses also contribute to the diversity of processes within the virocell providing new information that might eventually become part of the cell genome (Forterre, 2010). In this respect, non-coding RNAs may represent a suitable target for viral modulation in view of their viral origin and the variety of cellular processes they control (Witzany, 2009). In order to analyze a process of viral population variability influenced by the environment we worked on a system consisting of herpes simplex virus (HSV) and cell cultures in an environment containing sulfated polysaccharides known as carrageenans (CGNs). Cell heparan sulfate-like chemical structures in the CGNs are known to be very active and selective compounds against HSV (Carlucci et al., 1999). Their mechanism of action mainly affects viral adsorption stage, interacting with the surface glycoproteins, thus blocking interaction with cell receptors. Multiplication of HSV in the presence of CGN leads to the emergence of syn variants with phenotypic characteristics quite different from parental virus (Mateu et al., 2011; Artuso et al., 2016).

## Experimental Model

**FIGURE 1:**
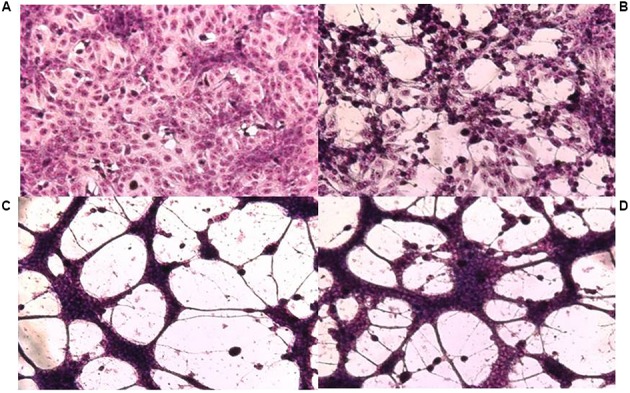
Cytopathic effect on Vero cells of HSV-1 F and its syncytial variants 48 h post-infection. **(A)** Uninfected control cells, **(B)** parental strain F, **(C)** passage 14, **(D)** passage 17. Cell monolayers were infected with the different viral variants at m.o.i: 0.1. The cells were fixed with methanol and stained with Giemsa 48 h p.i. (x100).

Isolation of viral variants was performed after successive passages of HSV in Vero cells subjected to increasing doses of CGN. For this purpose we monitored the changes of viral yield for each passage with or without CGN, using virus passages in the presence of acyclovir (ACV), the antiviral currently in use for herpetic infections, as controls. Also, the resistance pattern generated by the CGN, compared with ACV, has been evaluated. For CGN and ACV the initial doses were below the inhibitory concentration 50% (IC_50_) and were increased slightly in next passages (Carlucci et al., 2002). Titers of virus without CGN ranged from 10^7^ to 5 × 10^8^ PFU/ml throughout the 20 passages analyzed. Titers of virus in the presence of the polysaccharide was similar to the untreated control except for passages #1, 8, 14, and 19 where a 1.5 to 2 log drop in virus titer was detected. In the case of ACV titers were similar to untreated controls during the six passages analyzed after which recovered virus proved to be resistant to the antiviral. IC_50_ increased very rapidly in the first passages and the relative resistance also increased significantly from passage #4 onward, with a value of 46.6 μg/ml reaching 60.0 μg/ml in passage #6. In accordance to previous reports, selection of resistant virus to ACV was detected after few passages in the presence of the drug (Mateu et al., 2017). In the case of CGN, from passage 11 onward, the traditional type of cytopathic effect (CPE) of HSV, characterized by cell rounding and clumping that appeared as small focuses on the monolayer and eventually spread over the entire culture changed to the appearance of multinucleated cells (syncytia) due to the fusion of adjacent infected cells (**Figure 1**). Also, in this passage, a marked change was detected in the size of the viral plaques, coexisting small viral plaques (1 mm diameter) (similar to the parental strain) and large viral plaques of 1.5–2 mm diameter, until passage 16, when only large plaques could be observed. The augment in plaque size precedes the formation of syncytium. These changes in CPE were not detected after sequential passaging of HSV in the absence of CGN. IC_50_ also showed to be variable with a relative resistance (RR is the ratio between IC_50_ for each syn variant and IC_50_ for the F parental strain) between 1.5 and 6.6 for passages with CGN, while for viral controls without CGN the RR ranged between 1.6 and 3.4 (**Table 1**) (Carlucci et al., 2002). From passages 11 to 15 phenotypic changes (RR and CPE) in virus collected from supernatants showed a marked variability and, when passaged in the absence of CGN, recovered the phenotype of parental virus.

**Table 1:** Characteristics of viral cytophatic effect and drug susceptibility arising after different passages with CGN.

N° passage	IC_50_ (RR^a^)	Non syn (%)	Syn (%)
0	1.1 (1.0)	100	0
11	1.6 (1.5)	58.8	41.2 (rev.)^∗^
12	4.3 (3.9)	89.2	10.8 (rev.)
13	4.1 (3.7)	72.2	27.3 (rev.)
14	6.8 (6.2)	70.0	30.0 (rev.)
15	7.3 (6.6)	71.7	28.3 (rev.)
16	3.1 (2.8)	0	100 (irrev.)

*^∗^rev.: reversible, irrev.: irreversible. a = Relative Resistance.*

From passage 11 onward, the modification of the CPE accompanied by the increase of RR observed in the next passages suggest a successful adjustment of the viral population to the environment (CGN). It is tempting to speculate that treatment with CGN increases the “fluxus” of information in the viral population leading to the onset of a “temporary memory” as a useful tool for the virus to cope with environmental changes.

## Carrageenan and HSV

Evidence published in Meckes and Wills (2008) showed that like viruses can modulate cell signaling pathways when their receptors bind to the plasma membrane (Marsh and Helenius, 2006), also the signals can be transmitted in reverse through the envelope into any virus after receptor binding (Rein et al., 1994; Aguilar et al., 2003; Murakami et al., 2004; Wyma et al., 2004; Meckes and Wills, 2008). Interaction of HSV glycoproteins with its initial cell attachment protein (i.e., heparin or CGNs, a surrogate for heparan sulfate) triggers a rapid and highly efficient change in the structure of proteins of the tegument, a region between the viral membrane and the DNA-containing capsid. This phenomenum, has been described during studies of UL16. This protein associates with cytoplasmic capsids while on the other hand, interacts with a membrane-bound tegument protein, UL11.

The initial binding of HSV occurs through the interaction of glycoprotein gC and the cell receptor. As gC has a short cytoplasmic tail preventing signal transmission within the virion, probably, the interaction with other glycoprotein such as gB and gD may be necessary. On this respect, Cocchi et al. (2004) proposed a tripartite structure for the complex formed by gD together with gB and gH-L and its receptor and, one or various of the fusion glycoproteins. This complex would play an important role in recruiting/activating the fusion glycoproteins, activating them and promoting fusion of the viral envelope with cell membrane (Cocchi et al., 2004). But if this activation/deactivation of the glycoproteins are not coordinated with an eventual cellular fusion, as would be the case of the interaction CGN-virus, a “temporary memory” may be generated in the viral population. This memory would be crucial, particularly for DNA viruses, whose genomes are not prone to accumulate mutations in a fast manner as may be the case for RNA viruses, and may account for the RR and syn phenotype of virus collected during passages #11 to 16.

In view of the facts commented above, we may hypothesize that the binding of HSV through the glycoproteins gC, gB, gD to the CGN, leads to a structural change in the tegument proteins UL11 and UL16. Both proteins would interact with the viral capsid modulating the expression of immediate early genes (IE) (alpha) (Meckes and Wills, 2008; Svobodova et al., 2011) and, in consequence also modulating the remaining genetic blocks of herpes (beta and gamma).

Another point to address related to proteins of the viral tegument is linked to the fact that the variants manifested their syn CPE at a shorter time (16 h p.i.) than the parental virus (24 h p.i.). This observation may be related to the organization of the microtubules. Stable microtubules (MT) formation would be reduced in cells infected with syn variants by the viral Ser/Thr kinase, Us3 (Purves et al., 1987; Ryckman and Roller, 2004). Many viruses are dependent on MTs for their intracellular movement. During the early steps of infection, HSV is able to disrupt the centrosome, impairing MT organization. On the other hand, as infection goes further, HSV-1 induces the formation of stable structures formed stable MT subsets through inactivation of glycogen synthase kinase 3 beta by the viral Ser/Thr kinase, Us3 (Naghavi et al., 2013).

RNA network, particularly microRNAs (miRNAs), would be also modulated by the tegument proteins. miRNAs are small non-coding RNAs that interact with highly conserved proteins and are important in rapid gene regulation. miRNAs encoded by viruses exploit RNA silencing for regulation of their own genes, host genes, or both (Sullivan and Ganem, 2005). Also, viral miRNAs modulate biological processes of paramount importance: latent and lytic infection, evasion from the immune system, modulation of apoptosis, synthesis of viral macromolecules, etc. (Sullivan and Ganem, 2005; Boss and Renne, 2010). miR-H6 affects negatively the expression of ICP4. This protein is necessary for an efficient transcription of viral genes and regulates the onset of the characteristic CPE of HSV (Taylor et al., 2002; Umbach et al., 2008; Duan et al., 2012). On this line, miR-H2 targets ICP0 protein, an IE gene that has a major role in lytic infection and entrance of HSV into cells (Piedade and Azevedo-Pereira, 2016). On the other hand, miR-92944 is involved in the growth of virus and variants lacking miR92944 exhibited significant reductions in viral titers and fourfold reduction in plaque size (Munson and Burch, 2012). miR-23a and miR-146a are miRNAs of cellular origin that are involved in HSV replication because they interfere with the innate immune response diminishing the levels of interferon and activating pro-inflamatory cytokines (Ru et al., 2014). On the other side, HSV-1 induces the pro-inflammatory miR-146a. This molecule targets complement factor H and induces key elements of the arachidonic acid cascade (Hill et al., 2009). Also, it is an NF-κB-dependent gene, which in turn actively participates in the onset of the innate immune system (Taganov et al., 2006; Baltimore et al., 2008; Hill et al., 2009). Although these miRNAs are of cellular origin it cannot be ruled out that they are incorporated within the viral structure, providing the virus with valuable information for the next multiplication cycle in the presence of CGN.

In view of the facts exposed above, we hypothesize that the appearance of the syn variants during the early passages in the presence of CGN might be a consequence of an alteration of the tegument proteins which in turn modulate microtubules physiology and functioning of the RNA network in the virocell.

## Conclusion and Perspectives

The first inkling that herpesviruses modify cellular membranes was based on the observations that mutants differ wild type strains with respect to their effects on cells (Ejercito et al., 1968). These observations led the prediction that herpesviruses alter the structure and antigenicity of cellular membranes, a prediction fulfilled by (a) the demonstration of alterated structure and antigenic specificity and (b) the presence of viral glycoproteins in the cytoplasmic and plasma membranes of infected cells (Roizman and Sears, 1991). It’s know that the presence of gD in the plasma membrane of infected cells precludes reinfection of cells with the progeny virus released from that cell (Campadelli-Fiume et al., 1988). In our system the CGNs would act to interfere with viral glycoproteins of both virus and those exposed at the level of the cell membrane. The first phenotypic effect observed by the constant action of CGN with the virus is the increase in plaque size and the subsequent appearance of syn effects and variability in RR. We can assume that these glycoproteins could perceive the presence of the CGN in the environment and transmit this information both, inside the virus and the cell. In this sense, it has been shown the CGNs do not possess virucidal activity and have no action by pretreatment of the infected cell and do not penetrate into the cells (Carlucci et al., 1997, 1999, 2002; Yermak et al., 2012).

Likewise, herpesviruses are examples of dynamic and complex systems based on the interactions of multiple cellular and viral factors, leading to lifelong viral infections. These interactions control the expression of cellular proteins that may modulate the infection. In this work we modified the networks of target transcripts in the virocell by action of the CGN and verified a rapid viral adaptation to the presence of the polysaccharide before the manifestation of genetic modifications. We hypothesized that glycoproteins would fulfill, in addition to the functions already known, a fundamental function primarily as antennas of environmental perception. Host miRNA modulates viral infections by influencing antiviral responses, promoting several phases of the viral life cycle, or participating in cellular tropism. Also, as cellular miRNAs participate in multiple processes, their sequestration by the virus may cause a desregulation in the expresion of different cellular mRNAs, which might eventually lead to an aberrant process of protein translation. It is believed that one of the multiple parameters that cooperate to viral adaptation arises as a consequence of altered host miRNA–mRNA interactions, thus favoring the cellular environment for viral persistence or chronicity (Bruscella et al., 2017). Because viral miRNAs generally have a surprising lack of evolutionary conservation, it could be hypothesized that they are sites of rapid evolution, even as a driver of speciation (Kincaid and Sullivan, 2012). In agreement with Li et al. (2014), …“*viral RNAs could act as sponges that can sequester endogenous miRNAs within infected cells and thus impact the stability and translational efficiency of host mRNAs with shared miRNA response elements*”…(Li et al., 2014). Also, the use of miRNA as elements of shared responses between viral RNAs and host mRNA form complex networks during infection which affect replication, pathogenesis, and viral persistence. In this way the field of action of RNAs and viral mRNA would not only be limited to the level of viral protein synthesis or as PAMPs in innate immunity but would have multiple ways of working (Li et al., 2014). Finally, an important feature not to forget is that viral populations are plastic and in constant change. On this line, we are analyzing the virus recovered during the different passages with CGNs by High Throughput Sequencing in order to determine the relative abundance of viruses that exhibit differences with the parental strain at the genomic level.

We live neither in an arbitrary world of pure chance nor in a deterministic world without novelty and creativity. Life and Nature interplay in a never-ending process of evolution. Nature and humanity are interwoven creatively in this process, recognizing the “sensitive intelligence” of the viral population with the environment would be part of our learning.

## Author Contributions

All authors contributed to planning, writing, and revision of the manuscript. All authors read and approved the manuscript.

## Conflict of Interest Statement

The authors declare that the research was conducted in the absence of any commercial or financial relationships that could be construed as a potential conflict of interest.
